# Ovarian Cancer Cell Line Panel (OCCP): Clinical Importance of *In Vitro* Morphological Subtypes

**DOI:** 10.1371/journal.pone.0103988

**Published:** 2014-09-17

**Authors:** Corine M. Beaufort, Jean C. A. Helmijr, Anna M. Piskorz, Marlous Hoogstraat, Kirsten Ruigrok-Ritstier, Nicolle Besselink, Muhammed Murtaza, Wilfred F. J. van IJcken, Anouk A. J. Heine, Marcel Smid, Marco J. Koudijs, James D. Brenton, Els M. J. J. Berns, Jozien Helleman

**Affiliations:** 1 Department of Medical Oncology, Erasmus MC Cancer Institute, Rotterdam, The Netherlands; 2 Center for Biomics, Erasmus MC, Rotterdam, The Netherlands; 3 Functional Genomics of Ovarian Cancer Laboratory, Cancer Research UK Cambridge Institute, University of Cambridge, Li Ka Shing Centre, Cambridge, United Kingdom; 4 Department of Medical Oncology and Center for Personalized Cancer Treatment, University Medical Center Utrecht, Utrecht The Netherlands; 5 Molecular and Computational Diagnostics Laboratory, Cancer Research UK Cambridge Institute, University of Cambridge, Cambridge, United Kingdom; Peter MacCallum Cancer Centre, Australia

## Abstract

Epithelial ovarian cancer is a highly heterogeneous disease and remains the most lethal gynaecological malignancy in the Western world. Therapeutic approaches need to account for inter-patient and intra-tumoural heterogeneity and detailed characterization of *in vitro* models representing the different histological and molecular ovarian cancer subtypes is critical to enable reliable preclinical testing. There are approximately 100 publicly available ovarian cancer cell lines but their cellular and molecular characteristics are largely undescribed. We have characterized 39 ovarian cancer cell lines under uniform conditions for growth characteristics, mRNA/microRNA expression, exon sequencing, drug response for clinically-relevant therapeutics and collated all available information on the original clinical features and site of origin. We tested for statistical associations between the cellular and molecular features of the lines and clinical features. Of the 39 ovarian cancer cell lines, 14 were assigned as high-grade serous, four serous-type, one low-grade serous and 20 non-serous type. Three morphological subtypes: Epithelial (n = 21), Round (n = 7) and Spindle (n = 12) were identified that showed distinct biological and molecular characteristics, including overexpression of cell movement and migration-associated genes in the Spindle subtype. Comparison with the original clinical data showed association of the spindle-like tumours with metastasis, advanced stage, suboptimal debulking and poor prognosis. In addition, the expression profiles of Spindle, Round and Epithelial morphologies clustered with the previously described C1-stromal, C5-mesenchymal and C4 ovarian subtype expression profiles respectively. Comprehensive profiling of 39 ovarian cancer cell lines under controlled, uniform conditions demonstrates clinically relevant cellular and genomic characteristics. This data provides a rational basis for selecting models to develop specific treatment approaches for histological and molecular subtypes of ovarian cancer.

## Introduction

Epithelial ovarian cancer is the most lethal gynaecological malignancy in the Western world and advanced disease remains incurable for the majority of patients. Despite testing of diverse treatment strategies and new cytotoxic agents, optimal primary therapy and survival rates have not substantially changed since the introduction of platinum and taxane treatment [1]–[3].

Recent insights have indicated that ovarian cancer is a collective term for invasive pelvic cancers that are derived from different tissues with distinct histological and epidemiological features. The five major histiotypes have been shown to have specific genetic profiles and should be treated as distinct diseases. High-grade serous (HGS) carcinoma represents 80% of ovarian cancers and is defined by *TP53* mutation (96%), homologous recombination DNA repair defects (∼50%), *CCNE1* amplification and genomic instability [4]–[6]. By contrast, low-grade serous carcinomas are *TP53* wildtype and show frequently activating RAS pathway mutations [7], [8]. The remaining histiotypes are mucinous, endometrioid and clear cell [3]. Activating RAS pathway mutations are found in ∼40% of the mucinous tumours while endometrioid and clear cell tumours have *PIK3CA* (PI3Kinase component, 12%, 31% respectively), and *ARID1A* mutations (30%, 46%, respectively) [4], [5], [9], [10].

Furthermore, large studies have identified several molecular subtypes of HGS based on gene and microRNA expression [4], [11]. These subtypes suggest associations with specific biological processes (such as reactive stroma, mesenchymal, immunoreactive and proliferation) and poor prognosis, for example in the C1 stromal-subtype identified by Tothill et al. [4], [11]. Further exploring the clinical characteristics of these biological different subtypes and identifying an optimal treatment could identify new therapeutic strategies.

It is imperative that experimentally tractable in vitro models, such as cell lines, accurately represent the different histological and molecular subtypes in order to test new subtype-specific treatment strategies. Worldwide, there are about 100 ovarian cancer cell lines generated as described in literature [12]–[17] and about 70 of these are available at ATCC, ECACC, RIKEN, DSMZ, JCRB or Cancer Research Technology. Since 1990, an average of approximately 100 papers/year are published that rely upon ovarian cancer cell lines as a model. A major limitation for these studies is the fact that for most of ovarian cell lines their origin is poorly defined and owing to inadequate characterization it is not known which distinct histological or molecular subtype is represented.

We here describe an extensive and uniform characterization of a collection of 39 ovarian cancer cell lines commonly used for *in vitro* studies. We identified histological as well as morphological subtypes which associate with clinical pathological characteristics of ovarian carcinomas as well as prognosis. Furthermore, the morphological subtypes associate with the molecular subtypes identified by Tothill et al. [11]. In summary, these results can serve as a guide to select appropriate cell lines representing different histological and molecular subtype of ovarian cancer for *in vitro* therapeutic studies.

## Materials and Methods

### Cell lines and culturing

Cell lines studied were CAOV3, CAOV4, ES-2, OV-90, OVCAR3, TOV-112D, TOV-21G, UWB1.289, UWB1.289+BRCA1 (purchased from ATCC), 59M, A2780, A2780CIS, A2780ADR, ‘COLO720E’, COV318, COV362, COV362.4, COV413A, COV413B, COV504, COV644, OAW28, OAW42, OV56, OV7, OV17R, PEA1, PEA2, PEO1, PEO4, PEO14, PEO16, PEO23, SKOV3 (purchased from the European collection of cell cultures, ECACC via Sigma), 2774, A2780, HOC7, SKOV3, SKOV6 (courtesy of Gunter Daxenbichler, Dept. of Obstetrics and Gynaecology, University of Innsbruck, Austria), IGROV1 (courtesy of Dr. Irene Hamelers, Institute of Biomembranes, Utrecht University, The Netherlands). Table S1 summarizes the original source of the cell lines. The cell lines A2780 and SKOV3 were both obtained from an academic laboratory and also purchased from ECACC, and are described here as ‘A2780 ECACC’ and ‘SKOV3 ECACC’. Thirty-nine of the 40 cell lines were grown as monolayers, except for the semi-adherent cell line ‘COLO720E’ for which floating and attached cells were passaged. Attached cells were fully disaggregated by trypsinization between passages. The cell lines were maintained at 37°C in an incubator with humidified air with 5% CO2.

All cell lines were initially cultured using the medium and supplements as recommended by the suppliers. In contrast to other studies, we cultured all cell lines under the same culture conditions to avoid biases due to varying concentrations of supplements within different media (e.g. growth factors) or different percentages of serum. All cell lines were cultured in RPMI-1640 glutamax, Gibco/Invitrogen supplemented with 10% FCS (Lonza, source: Brazilian, Lot nr 1SB002), 100 U/ml penicillin, 100 µg/ml streptomycin, 50 µg/ml gentamycin. All experiments and nucleic acid isolations were performed after culturing the cell lines for at least one month using these standard conditions.

UWB1.289 is a BRCA1-null cell line from woman with a germline *BRCA1* 2594delC mutation. UWB1.289+BRCA1 is stable transfected with a pcDNA3 plasmid containing a *BRCA1* insert and was maintained with 200 µg/ml Geneticin (G418) to maintain selection for transfected cells. A2780ADR and A2780CIS were challenged once a month with 100 nM Doxorubicin and 1 µM Cisplatin respectively.

### Short tandem repeat analysis

The PowerPlex 16 System (Promega) was used according to the manufacturer's protocol using an ABI PRISM 3100 to generate an STR (Short tandem repeat) fingerprint of each cell line to determine unique identity and/or lack of co-culture contamination. In addition, the STR profile was checked before and during culturing of the cells to ensure that cross-contamination or erroneous substitution did not occur.

### Growth curves and drug sensitivity assay

The MTT colorimetric assay, which measures metabolic activity of viable cells, was used to generate growth curves and determine the chemosensitivity of the cell lines.

First, growth curves were generated in duplicate to determine the optimal number of cells to use for the drug sensitivity assay. This was done to avoid growth inhibition due to seeding not enough cells or depletion of the medium after seeding too many cells. The highest number of cells showing continuing exponential growth after five days was selected for the drug response MTT assays (Table S1, File S1).

Response curves were generated for Carboplatin, Cisplatin, Oxaliplatin, Doxorubicin and 5-Fluorouracil (Intravenous solutions, Pharmachemie, The Netherlands), Paclitaxel (Intravenous solution, Ebewe Pharma, Austria), Docetaxel (dissolved in DMSO, Sigma), and Gemcitabin (for intravenous use, dissolved in PBS, Sun Pharmaceutical Industries Europe BV, The Netherlands). Cell viability was assessed in quadruplicate using the MTT assay after a five day exposure to 18 concentrations of the compound. Phoenix WinNonLin 1.1 software (Pharsight) was used to fit a dose response curve and to calculate the 50% growth inhibition values (GI50) with error and 95% confidence intervals (see also File S1).

### DNA and total RNA isolation

The NucleoSpin kit (Machery-Nagel) was used according to the manufacturer's protocol to isolate DNA from cells harvested with trypsin and washed with PBS.

For the isolation of total RNA (including microRNAs), exponentially growing cells were directly lysed in culture flasks with RNA-Bee to avoid changes in expression due to harvesting or washing. Total RNA was isolated from lysates as previously described [18]. Three independent isolates of RNA from each line were pooled for mRNA and microRNA expression analysis to decrease possible bias from outlier values.

### microRNA and gene expression analysis

For microRNA expression analysis, TaqMan Array Human MicroRNA A fluidic Cards v2.0 (Applied Biosystems) containing qRT-PCR assays to quantify 381 unique microRNAs were used according to the manufacturer's protocol. The expression data was normalized using the median Ct of all measured microRNAs as described by Vandesompele et al. [19] (see also File S1). The normalized expression data for all 384 microRNAs is given in Table S2.

Gene expression was measured using the GeneChip Human Exon 1.0 ST Array (Affymetrix) according to manufacturer's protocol (see also File S1). The acquired expression data was pre-processed using the Robust Multichip Analysis (RMA) algorithm within the Affymetrix Expression Console software that performs background correction, normalization and probe set summarization per transcript resulting in one expression value per gene. The gene expression data has been deposited in the Gene Expression Omnibus (GSE53418).

### SNaPshot mutation analysis

SNaPshot analysis was performed as described previously [20]–[22] using an Applied Biosystems SNaPshot Multiplex Kit. The nucleotide and corresponding amino acid changes evaluated were for PIK3CA, BRAF, HRAS, NRAS and KRAS are listed in the supplement methods (*File S1*).

### Mutation and amplification analyses with SOLiD exon sequencing

Processing of samples for sequencing on the SOLiD5500 sequencing platform (Life Technologies) was performed on a Sciclone NGS liquid handling robot (Perkin Elmer), based on the protocol for manual library preparation [23]. Sure-Select enrichment for genes of interest was performed in a multiplexed fashion of up to 16 samples per reaction (based on Nijman et al. [24]) using a custom designed enrichment kit.

Data were mapped to the reference genome (GRCh37/hg19) using BWA and SNP and indel calling was done using a custom analysis pipeline. Based on the COSMIC database [9] and the review by Berns et al. [5], 53 genes that are often mutated or amplified in ovarian cancer were selected for analyses (see supplement methods in File S1 for the list of genes).

For the analysis of gene amplification, robust Z-scores were calculated per exon as described by Iglewicz and Hoaglin [25]. A gene is amplified with a Z-score greater than 2 and highly amplified if greater than 3.

The SOLiD Exon sequencing has been deposited in the European Nucleotide Archive (PRJEB5114).

### Mutation analysis by Tagged-amplicon deep sequencing (Tam-Seq)

The coding sequences of TP53, BRCA1, BRCA2, PTEN, PIK3CA, EGFR and APC genes were amplified and sequenced using the Tam-Seq method and the Fluidigm Access Array 48.48 (Fluidigm CA, USA) according to manufacturer's protocol as described previously [26]. Sequence analysis and variant verification was performed as described previously [26], [27]. The primer sequences are available upon request. The Tam-Seq data has been deposited in the European Nucleotide Archive (PRJEB5183).

### Microsatellite instability

Microsatellite instability was determined using the MSI Analysis System Version 1.2 (Promega) according to the manufacturer's protocol and as performed routinely electrophoresis using an ABI PRISM 3100. MSI is determined on five mononucleotide repeat markers (BAT-25, BAT-26, NR-21, NR-24 and MONO-27).

### FACS analyses

The cells were harvested and stained with properly titrated fluorochrome-conjugated monoclonal antibodies (as described before [28]). In brief, cells were stained with antibodies against luminal cytokeratin's (CK) conjugated to PE (mixture of cytokeratin 8, 18 -clone C11- and cytokeratin 19 -clone A53-B/A2-, Veridex LCC, Raritan, NJ), CD24-FITC (clone SN3, eBiosciences, San Diego, CA), CD44-PeCy7 (clone IM7, eBiosciences) and EpCAM-FITC (clone EBA-1, BD Biosciences, San Jose). CK staining was preceded by a fixation and permeabilization step using FIX & PERM reagents (An Der Grub Bio Research GmbH, Austria). Protein expression was measured on a FACSCanto flow cytometer (BD Biosciences). The signal-to-noise ratio (s/n) was calculated (i.e. geometric mean fluorescence intensity (MFI) of the stained population divided by the MFI of the unstained population) to correct for auto fluorescence.

### Statistics and visualization

Statistical analyses were performed on the 33 cell lines that were generated from unique ovarian cancer patient material. Duplicates (SKOV3 and A2780), *in vitro* derivatives (A2780ADR, A2780CIS, COV362.4 and UWB1.289+BRCA1) and the possibly contaminated cell line ‘COLO720E’ were left out of these analyses. Statistical analyses were done using nonparametric methods within the STATA statistical package, release 12.0 (STATA Corp., College Station, TX). P-values were two-sided and P<0.05 was considered statistically significant.

BRB array tools (v4.3.1) was used to select genes and microRNAs differentially expressed between the three morphological subtypes. First, microRNAs with >80% missing values were filtered out. Then missing values that were not flagged due to low quality were replaced with the minimal value over all microRNAs and samples followed by 2log normalization. Within BRB, the 50% most variable microRNAs and mRNAs were selected and the class comparison algorithm was used to calculate the permutation p-value after 10.000 permutations. P<0.05 was considered significant. Hierarchical clustering was done on median centred mRNA and microRNA expression data using Eisen Gene cluster 3.0. For the GSE9891 data set multiple probes per gene were averaged which most closely resembles the approach taken by the exon arrays (i.e. the probe set summarization within the Affymetrix Expression Console software). Next, the cell line gene expression data and the GSE9891 data (excluding the LMP samples) were median centred separately to correct for differences between the platforms used and subsequently combined before the hierarchical clustering. Identification of biological function of the morphology related genes was performed using IPA (v16542223, Ingenuity Systems).

## Results

**Figure 1:**
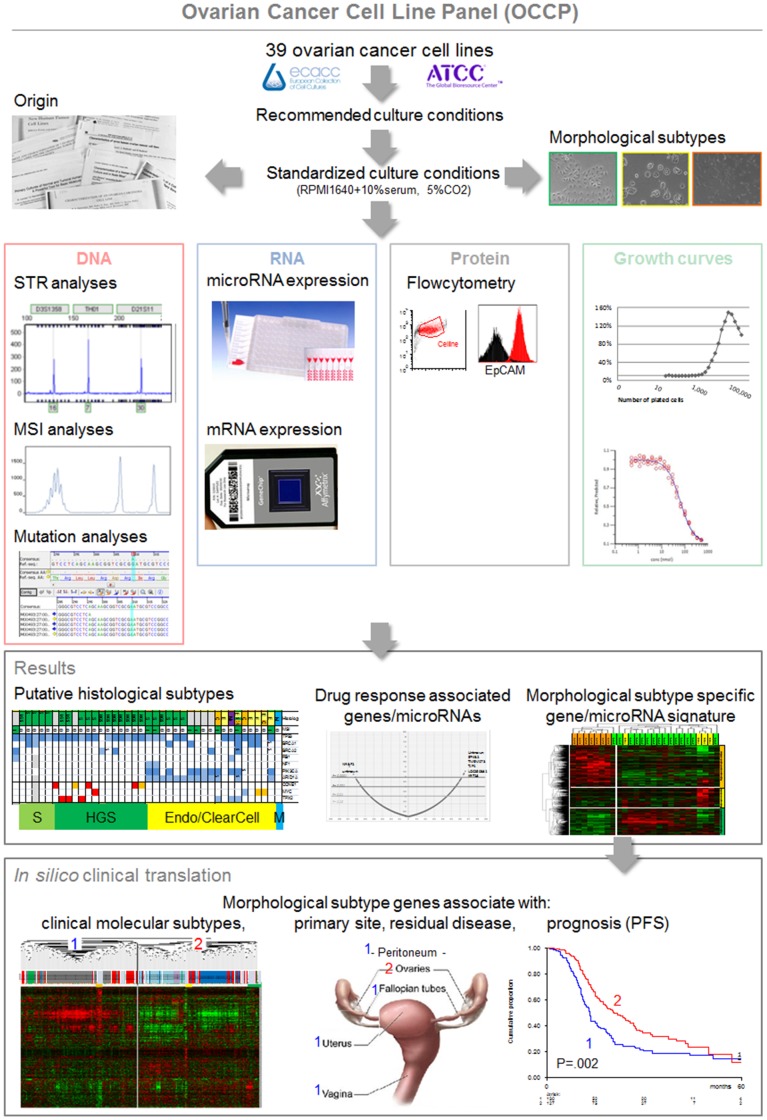
Overview of the presented data, results and *in silico* clinical translation. *STR short tandem repeat, MSI microsatellite instability, PFS progression-free survival*.

Figure 1 gives a summary of the experimental plan including culturing all cell lines with the same culture conditions to avoid biases due to varying concentrations of supplements within different media (e.g. growth factors) or serum.

### Short tandem repeat (STR) analysis

The measured number of repeats for each of the 16 STR loci as well as reference STR profiles from public databanks and literature are given in Table S3.

The concordance between the peaks of the reference profiles and the profiles generated in this study was 100% for 22 cell lines, 90–99% for ten cell lines and 79–89% for four cell lines. For three cell lines, there are no references available (Table S3).

One cell line, ‘COLO720E’, showed only 4% concordance with the STR profiles published by Korch et al. [29]. COLO720E has been described to be mixture of the COLO684 and COLO685 cell lines which are both derived from uterine adenocarcinoma [30]. ‘COLO720E’ had two TP53 mutations described (c.1118del1, c.C413T), supporting the possibility of two cell populations. However, these two TP53 mutations were recently reported by Anglesio et al. although their COLO720E cell line did match the publicly available STR reference [31]. Since ‘COLO720E’ has been recently retracted from the ECACC cell line repository, we excluded it from further data analyses.

### Origin

**Figure 2:**
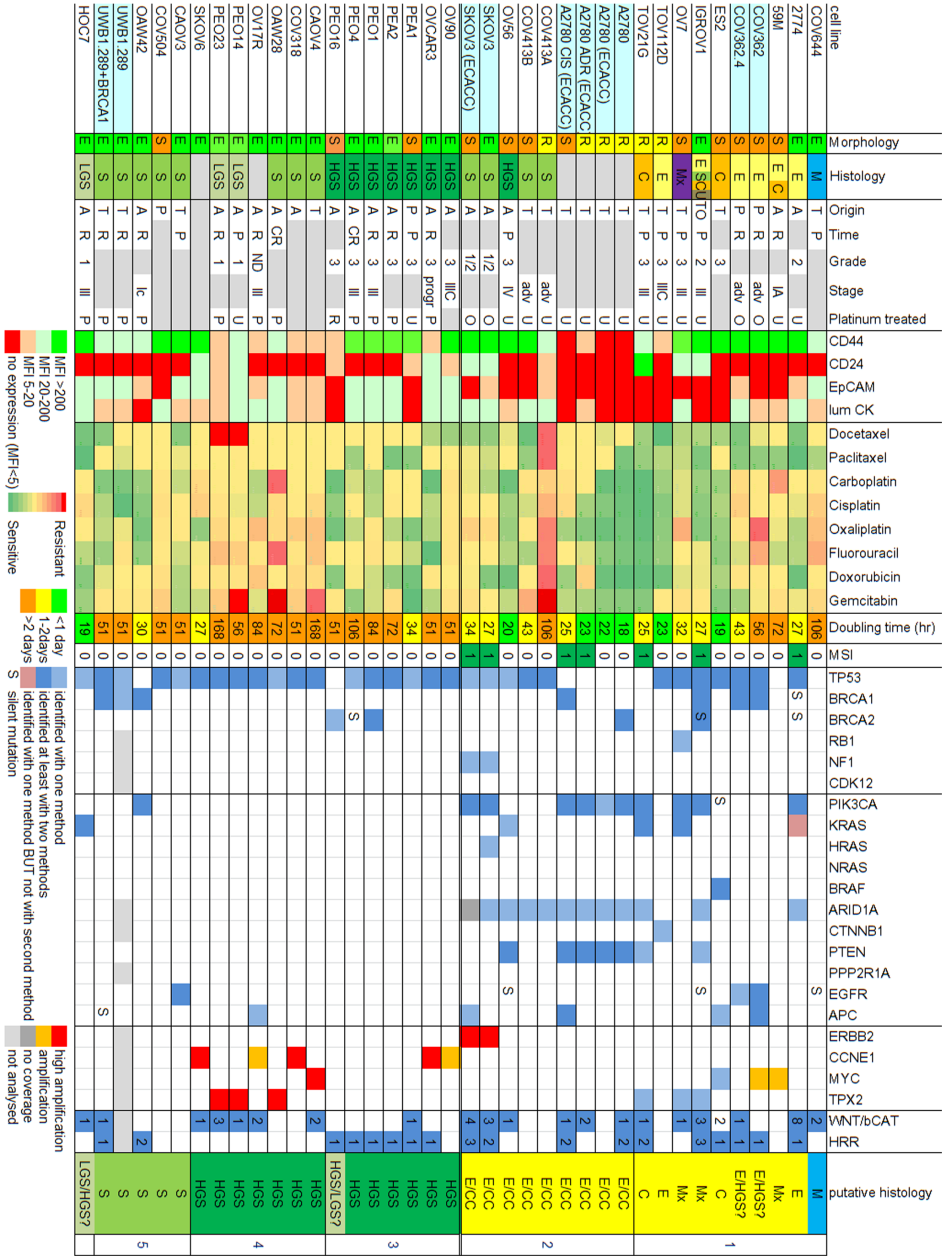
Overview of the cell line characteristics. *Morphology*: E Epithelial, R Round, S Spindle, *Histology & Putative Histology*: S serous, HGS high-grade serous, LGS low-grade serous, E endometrioid, C clear cell, Mx mixed, M mucinous. *Origin*: A ascites, T tumour tissue, TM tissue from metastasis, TO ovarian tumour tissue, P pleural effusion. *Time*: P primary disease, R relapsed disease, CR at clinical resistance. *Platinum treated*: U untreated, P platinum-based treatment, O other chemotherapy, R radiotherapy. *Protein markers*: bright red no expression (signal –to-noise ratio <5), light red low expression (signal –to-noise ratio 5–20), light green expression (signal –to-noise ratio 20–200), bright green high expression (signal –to-noise ratio >200), grey not determined. *Therapy response*: green to red scale sensitive to resistant. *Doubling time*: green less than one day, yellow 1–2days, orange >2days. *MSI* microsatellite instability. *Gene mutations*: dark blue identified by at least two methods, light blue identified by one method, light red identified with one method BUT not with second method. *Gene amplification*: orange amplified (2–3x SD above the median), red highly amplified (>3x SD above the median). The WNT/bCatenin pathway (WNT/bCAT) and homologous recombination repair (HRR) columns show the number of mutated genes in these pathways.

The available literature information on the origin of the 39 cell lines is summarized in Figure 2 (additional data in Table S1) [32]–[53]. Thirty-three cell lines were generated from unique patient material, not including the duplicate sources (SKOV3, A2780), in vitro derivatives (A2780ADR, A2780CIS, COV362.4, UWB1.289+BRCA1) and the possibly contaminated cell line ‘COLO720E’. Histology was described for 30/33 cell lines and showed a similar frequency distribution compared to ovarian carcinomas with the majority being serous (21/33) (Figure 2).

The origin (ascites, tumour tissue or pleural effusion), time (primary disease, recurrence, at clinical resistance) and whether the patient had received platinum-based chemotherapy before culture was known in 32, 21 and 25 cell lines respectively. The tissue-originated cell lines were mostly from platinum-naive patients (7/8, 88%) and primary disease (6/7, 86%) whereas the ascites-originated cell lines were more often platinum-treated (9/15, 60%) and cultured after relapse or clinical resistance (10/12, 83%).

### Microsatellite instability

Microsatellite instability for all five markers studied was observed for: 2774, both SKOV3 sources, IGROV1, TOV21G and ‘COLO720E’ (Figure 2). A2780ADR and A2780CIS showed instability for four of the five markers (NR-21, BAT26, NR-24, MONO-27) and were bi-allelic for the fifth marker BAT25. In contrast, the parental A2780 ECACC cell line (also from the European cell culture collection ECACC) showed only microsatellite instability for one marker (NR-21) and was also bi-allelic for BAT25. The second A2780 source from an academic laboratory showed no microsatellite instability (only a small shift for NR-21) but was also bi-allelic for BAT25. This indicates that different cultures of the same cell line can develop or select genetic differences as we described previously for different A2780 cell lines [54].

Although the numbers are very small, the four MSI cell lines showed significantly less concordance of their STR profile to the reference STR profile (Mann-Whitney U test, p<0.0013) and more mutations than the 29 non-MSI cell lines (median 13 versus 2 mutations per cell line, Mann-Whitney U test p<0.001).

### Morphological subtypes

Three morphological phenotypes were distinguished based on two independent observations of morphological and growth characteristics during culturing from low-density up to 70/80% confluence, i.e. cell shape, cell size and growth pattern during culturing as well as proliferation rate. Figure S1 shows representative images of six examples of each of the three morphological subtypes to illustrate differences in cell shape, size and growth pattern between the morphological subtypes. The ‘Epithelial’ morphological subtype was characterized by flattened epithelial-like cells that grew in sheets of regular or irregular clusters (n = 21). In the ‘Round’ subtype (n = 7), cell size was smaller with a round shape and high proliferative rate. Cells grew separately or as irregular clusters, adhered loosely to tissue culture dishes and often grew on top of each other. Cells with the ‘Spindle’ subtype (n = 12), were stretched and spindle shaped and grew as separate cells or irregular clusters.

There was an association between morphology and the origin of the 33 unique cell lines, 74% of Epithelial cell lines originated from ascites (14/19), all four Round cell lines were culture from tissue, while Spindle cell lines originated equally from ascites, tissue or pleural effusion (all 3/9, 33%) (Fisher's exact p = 0.002). Although not significant, Epithelial cell lines were more often of serous origin (83%) compared to Round (33%) and Spindle (56%) cell lines (Fisher's exact, p = 0.095).

Interestingly, the morphological subtype was significantly associated with prior platinum-based chemotherapy (10/14 Epithelial had previously received platinum-based treatment versus 4/4 Round and 7/7 Spindle untreated lines; Fisher's exact test, p<0.002).

### Protein markers

The expression of CD44, CD24, EpCAM and luminal cytokeratin's are given in Figure 2.

The stem cell marker CD44 was expressed in 31/33 of the cell lines and was associated with morphology (Kruskal-Wallis p = 0.0037). Interestingly, 6/9 Spindle cell lines showed high CD44 expression combined with absent CD24 expression which has been suggested as a characteristic of stem cell populations. By contrast, CD44^high^/CD24^−^ expression was not observed in the four Round cell lines and in only 6/20 Epithelial cell lines.

The epithelial markers EpCAM and luminal Cytokeratin's were expressed in most Epithelial cell lines (19/20 and 18/20 respectively) but only in one Round cell line (1/4). All Spindle cell lines showed absent or very low expression of EpCAM but 5/9 did express luminal Cytokeratin's. EpCAM and luminal Cytokeratin's expression was associated with morphology (Kruskal-Wallis, p<0.001 and p<0.024 respectively) as well as serous versus non-serous histology (Mann-Whitney, p = 0.004 and p = 0.108 respectively).

### Gene mutation and amplification

The mutation status of 53 genes was determined with three techniques: SNaPshot, SOLiD and/or Tam-Seq exon sequencing. Discordant data between these three techniques were checked manually using the raw sequence data. Table S4 lists all mutation data which is summarized in Figure 2. In total, we detected 178 mutations in 45 genes in all 39 cell lines. The most frequently mutated gene was TP53, mutated in 27/33 cell lines including 7/8 high-grade serous, 9/10 serous and unexpectedly in 3/3 low-grade serous.

We determined the amplification of CCNE1, MYC, TPX2 and ERBB2 by comparing the sequencing coverage for each exon to the median overall coverage [55]. Amplification was observed for CCNE1 (n = 5), MYC (n = 3), TPX2 (n = 3) and ERBB2 (n = 2) (Figure 2, Figure S2A-C). When we compared genomic amplification of each gene with mRNA expression, MYC and TPX2 were highly expressed in the majority of the cell lines including lines that showed amplification (data not shown). The cell lines showing *ERBB2* or *CCNE1* amplification showed the highest expression of these genes. For *CCNE1* this association was significant (Mann-Whitney p<0.001, Figure S2D).

### Response to chemotherapeutics

**Figure 3:**
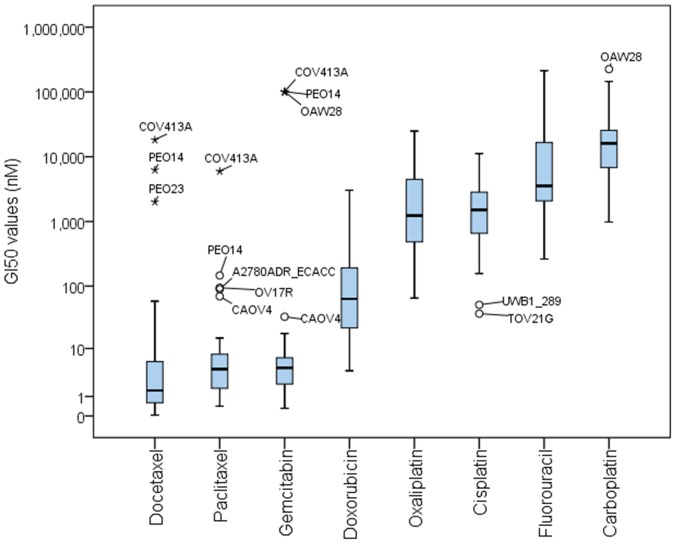
Box-plot of the concentration of drug causing 50% growth inhibition (GI50) for all 39 cell lines and eight therapeutics. *The whiskers extend to 1.5 times the height of the box (i.e. 25^th^ percentile to median and median to 75^th^ percentile) or, if there is no value in that range, to the minimum or maximum values*.

For all drugs, the concentration causing 50% growth inhibition (GI50 values) showed a two log range between sensitive and resistant cell lines (Figure 3). Notably, median GI50 values (nM) showed marked differences between drugs with the most widely separated compounds, Docetaxel and Carboplatin, having a 10000-fold difference (Figure 3).

Germline mutations in *BRCA1* and *BRCA2* have been associated with response to platinum drugs. However, there was no clear association between response to platinum and mutation status or expression of *BRCA1* and *BRCA2* in our experiments (data not shown). Approximately 50% of the BRCA mutations observed were not known to be clinically relevant (Table S4) and therefore might not affect BRCA function. However, the two cell lines with known germ-line mutations in BRCA1 (UWB1.289) and BRCA2 (PEO1) were relatively sensitive to Cisplatin.

The response for all drugs showed a significant positive correlation with the proliferation rate of the cell lines, i.e. the doubling time (Table 1).

**Table 1:** Association of chemotherapy response (concentration causing 50% growth-inhibition) with doubling time (T2, hours) and protein marker expression (s/n) in 33 unique ovarian cancer cell lines.

	*Docetaxel*	*Paclitaxel*	*Carboplatin*	*Cisplatin*	*Oxaliplatin*	*Fluorouracil*	*Doxorubicin*	*Gemcitabin*
T2	**0.663 (<0.001**)	**0.370 (0.034)**	**0.459 (0.007)**	**0.505 (0.003)**	**0.634 (<0.001)**	**0.742 (<0.001)**	**0.685 (<0.001)**	**0.548 (0.001)**
CD44	**−0.430 (0.013**)	**−0.347 (0.048)**	0.309 (0.080)	0.159 (0.378)	0.092 (0.610)	−0.109 (0.546)	−0.251 (0.158)	−0.212 (0.236)
CD24	0.095 (0.600)	0.128 (0.480)	−0.163 (0.365)	−0.038 (0.836)	−0.183 (0.308)	0.036 (0.844)	−0.004 (0.981)	0.035 (0.845)
EpCAM	0.177 (0.324)	0.108 (0.550)	0.214 (0.232)	**0.411 (0.017)**	0.252 (0.158)	0.342 (0.051)	0.187 (0.297)	0.083 (0.644)
lumCK	0.159 (0.378)	0.095 (0.599)	**0.391 (0.025)**	**0.392 (0.024)**	**0.541 (0.001**)	0.320 (0.069)	**0.369 (0.035)**	0.241 (0.176)

T2 doubling time, LumCK luminal cytokeratins, Spearman correlation and (p-value) are shown in bold when significant (p<0.05).

Table 1 shows the association between drug response and the expression of the protein markers CD44, CD24, EpCAM and Luminal cytokeratin's. Interestingly, cell lines with a high CD44 expression were more sensitive to taxanes. By contrast, cell lines showing high expression of luminal cytokeratin's were relatively resistant to the three platinum drugs tested, and Doxorubicin. High expression of EpCAM was also correlated with relative resistance to Cisplatin. Luminal cytokeratin and EpCAM are strongly correlated and absent expression of both markers was most commonly seen in Round cell lines that were in general very sensitive to most therapeutics. If the four Round cell lines are excluded, the only significant association observed was between Oxaliplatin response and expression of luminal cytokeratin's, indicating that this small group of cell lines is mainly causing other associations.

**Figure 4:**
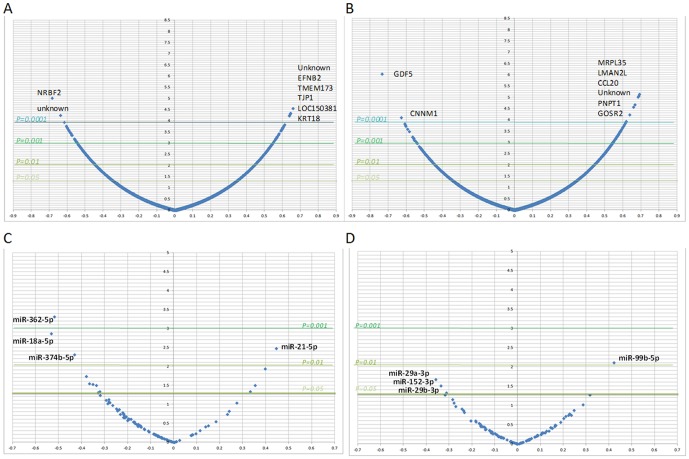
Volcano plot with the correlation between the GI50 values for Cisplatin (A&C) and Paclitaxel (B&D) and the expression of each mRNA (A&B) or microRNA (C&D). *X-axes spearman correlation, Y-axes -Log of the p-value.*

We next used the Spearman rank correlation to test for correlation between the GI50 values and the expression of each mRNA or microRNA in order to identify genes and microRNAs associated with response to therapy. The number of mRNAs associated with response to the eight therapeutics tested ranged from 22–310 genes (p<0.001) and 1–10 microRNAs (p<0.01). Figure 4 shows for each mRNA and microRNA the correlation and significance between its expression and response to Cisplatin and Paclitaxel.

### Putative histological subtypes

We designated cell lines as putative HGS or endometrioid/clear cell cell lines based on four criteria. Criteria for the putative HGS origin were: 1) HGS origin with TP53 mutation and no MSI, or 2) serous origin with TP53 mutation and amplification of CCNE1, MYC or TPX2. Criteria for putative endometrioid/clear cell origin were: 1) endometrioid and/or clear cell origin, or 2) ARID1A mutation.

Using these criteria, we deduced the putative histological subtype for the 39 cell lines as 20 non-serous (Figure 2, last column group 1&2) and 19 serous cell lines which included 14 high-grade serous and one low-grade serous line (Figure 2, last column group 3–5).

From the non-serous cell lines, group 1 (see also Figure 2), contains ten cell lines that were described to be non-serous and only in COV362 and 59 M, a MYC amplification contradicts this. Interestingly, group 2 includes 10 cell lines with an ARID1A mutation which is strongly associated with clear cell or endometrioid histology.

From the serous cell lines, group 3 contains seven cell lines described as high-grade serous (HGS) in origin and all, except PEO16, show a TP53 mutation as expected for HGS tumours. PEO16 does show a BRCA2 mutation and a TP53 mutation might have been missed since it was sequenced with one technique, although with a reasonable coverage (mean coverage  = 113). In addition, we identified seven other putative HGS cell lines (Group 4) that were previously described as (low-grade) serous or unknown histology that all show a TP53 mutation combined with an amplification of CCNE1, MYC or TPX2 which are characteristics of the HGS subtype.

The seven remaining putative serous cell lines, Group 5, were described to be of serous origin and six show a TP53 mutation but no additional HGS characteristics. OAW42 shows no TP53 mutation but a PIK3CA and a BRCA1 mutation and both SKOV3 cell lines are MSI which is more often seen in the mucinous and endometrioid subtypes [54]. The putative low-grade serous cell line, HOC7, was described to be of LGS origin and shows a KRAS but also a TP53 mutation which is not a LGS characteristic.

Interestingly, the putative histological subtypes (in contrast to the published histological tumour of origin) were significantly associated with the morphological phenotypes. Twelve out of 13 HGS cell lines have an Epithelial morphology and one Spindle, 4/6 serous cell lines have Epithelial and two Spindle morphology and for the 13 Clear cell/Endometrioid/Mixed cell lines three have Epithelial, four Round and six have a Spindle morphology (Fisher's exact p = 0.002).

### Molecular subtypes

Four molecular subtypes were defined by unsupervised clustering of the expression of the 80% most variable genes (n = 17609) and microRNAs (n = 177). The 80% most variable genes and microRNAs were selected by ranking based on their variability in expression across the 26 unique cell lines (excluding the seven PEO/PEA cell lines that were not profiled).

The four clusters identified associated with specific cell line characteristics (Figure S3). The putative histology HGS is enriched in the second cluster while the endometrioid/clear cell putative histology is enriched in the two right clusters. Clusters 1 and 2 contained slower growing cell lines with mostly Epithelial morphology, expression of EpCAM and luminal cytokeratin's, and showed more frequent gene amplifications and less MSI and non-synonymous mutations compared to clusters 3 and 4. Interestingly clusters 1 and 2 also contains more cell lines originating from ovarian cancer cells from platinum-treated patients suggesting that recurrences after platinum-based chemotherapy may contain more often slow cycling cells with epithelial characteristics and relative resistance to chemotherapeutics.

### Morphological subtype specific genes, microRNAs and pathways

**Figure 5:**
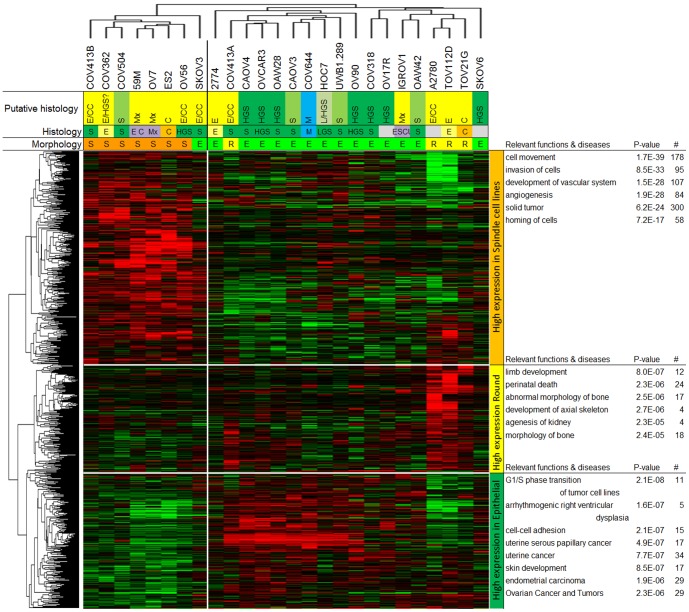
Hierarchical clustering based on the expression of the 1141 genes and 18 microRNAs differentially expressed between the three morphological subtypes. *Histology & Putative Histology*: S serous, HGS high-grade serous, LGS low-grade serous, E endometrioid, C clear cell, Mx mixed, M mucinous, *Morphology*: E Epithelial, R Round, S Spindle, *Red colour* high expression, *Green colour* low expression.

We selected 1141 genes and 18 microRNAs differentially expressed between the three morphological subtypes (permutation derived p<0.05) (Table S5). Hierarchical clustering using these differentially expressed genes on the 26 unique ovarian cancer cell lines resulted in two cell line clusters: a Spindle cluster and an Epithelial cluster that contained a distinct subcluster of three Round cell lines (Figure 5). In addition, three gene/microRNA clusters could be identified showing high expression in Spindle, in Round and in Epithelial cell lines (Figure 5). The top biological functions and diseases of these three gene/microRNA clusters are shown in Figure 5 together with the p-value and the number of genes/microRNAs associated with this function or disease.

Two cell lines did not cluster with their morphological subtype, SKOV3 clusters with Spindle instead of Epithelial (as is SKOV3 ECACC) and COV413A with Epithelial instead of Round.

### Morphological subtypes associate with clinical phenotype

**Figure 6:**
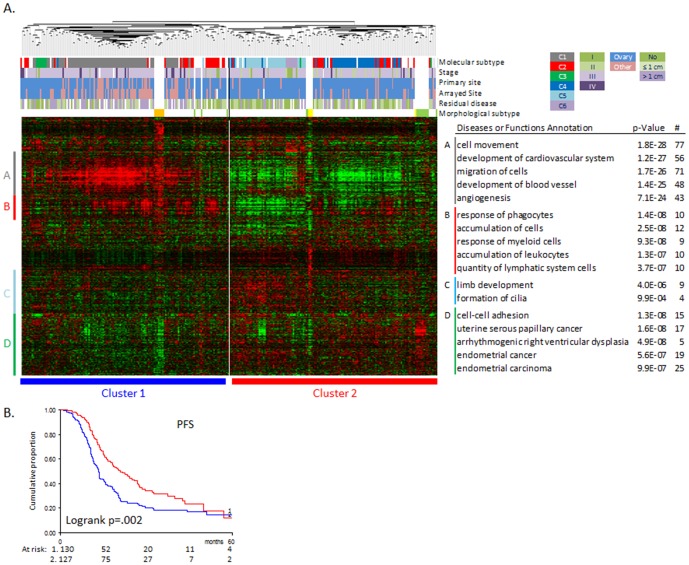
Hierarchical clustering of 26 cell lines and 267 ovarian carcinomas based on the expression of the 1141 morphological subtypes-associated genes (A) and the association with progression-free survival (B). The diseases and functions associated with gene clusters A-D are shown together with the p-value and the number of genes associated (#). *Red colour* high expression, *Green colour* low expression.

We used the expression data for 267 malignant ovarian carcinomas (GSE9891) [11] to determine the clinical significance of the morphological subtypes seen in our cell lines. After averaging of duplicate probes, the hierarchical clustering based on the expression of the 1141 morphological specific genes showed two tumour clusters, resembling 1) the Spindle-shaped cell lines and 2) the Epithelial and Round cell lines (Figure 6). Patient and clinicopathological features are given in Table 2 as well as their association with the two morphological clusters. The ‘Spindle-like’ tumour cluster 1 showed a significant enrichment for metastasis as arrayed and primary site, higher stage, sub-optimal debulking and decreased progression-free survival (Table 2, Figure 6). There was also a strong association with the six molecular subtypes identified by Tothill et al. [11]. Cluster 1 contains all C1 (stromal) and most C3 (LMP-like) carcinomas while (almost) all C4, C5 (mesenchymal) and C6 (low-grade endometrioid) tumours are within Cluster 2. The C2 (immune) carcinomas are evenly divided over the two morphological tumour clusters.

**Table 2:** Patient characteristics, clinicopathologic features and the molecular subtypes defined by Tothill et al. relative to the morphological clusters.

	*All patients*		*Morphological clusters*				
			Cluster 1		Cluster 2		p-value
Molecular subtype							
C1	83	(35.6%)	83	100.0%	0	0.0%	**<0.001** §
C2	50	(21.5%)	27	54.0%	23	46.0%	
C3	10	(4.3%)	8	80.0%	2	20.0%	
C4	46	(19.7%)	1	2.2%	45	97.8%	
C5	36	(15.5%)	1	2.8%	35	97.2%	
C6	8	(3.4%)	0	0.0%	8	100.0%	
Age at diagnosis							
Median	59		60		58		0.626£
Range	23–80		23–80		33–80		
Stage							
I	16	(6.0%)	6	37.5%	10	62.5%	**0.007** §
II	14	(5.2%)	2	14.3%	12	85.7%	
III	212	(79.4%)	109	51.4%	103	48.6%	
IV	21	(7.9%)	15	71.4%	6	28.6%	
Unknown	4	(1.5%)	-	-	-	-	
Histological subtype							
Serous	246	(92.1%)	129	52.4%	117	47.6%	**0.018** §
Endometrioid	20	(7.5%)	5	25.0%	15	75.0%	
Adenocarcinoma	1	(0.4%)	-	-	-	-	
Grade							
1 (low)	11	(4.1%)	7	63.6%	4	36.4%	0.548§
2	97	(36.3%)	46	47.4%	51	52.6%	
3 (high)	155	(58%)	80	51.6%	75	48.4%	
Unknown	4	(1.5%)	-	-	-	-	
Primary site							
Ovary	225	(84.3%)	103	45.8%	122	54.2%	**0.01** ¥
Fallopian tube	8	(3%)	32	76.2%	10	23.8%	
Peritoneum	34	(12.7%)					
Arrayed site							
Ovary	181	(68.3%)	71	39.2%	110	60.8%	**<0.001** §
Fallopian tube	3	(1.1%)	63	75.0%	21	25.0%	
Peritoneum	71	(26.8%)					
Other b	10	(3.8%)					
Unknown	2	(0.8%)	-	-	-	-	
Residual disease							
Nil macroscopic	68	(25.5%)	23	33.8%	45	66.2%	**0.004** §
≤1 cm	76	(28.5%)	39	51.3%	37	48.7%	
>1 cm	82	(30.7%)	50	61.0%	32	39.0%	
Macroscopic, size unknown	6	(2.2%)	-	-	-	-	
Unknown	35	(13.1%)	-	-	-	-	
Total	267		135	50.6%	132	49.4%	

§ Pearson Chi-square test,

¥ Fisher's Exact,

£ Two-sample Wilcoxon rank-sum (Mann-Whitney) test.

We also observed that the Spindle cell lines clustered with the C1 (stromal) tumours, the Round cell lines with the C5-mesenchymal subtype and the Epithelial cell lines with the C4 subtype (Figure 6). The clustering of the cell lines with the different tumour subtypes can be used to select relevant cell lines for tumour subtype specific studies.

## Discussion

Worldwide about 100 ovarian cancer cell lines are available and since 1990, a total of over 2000 papers have been published using these cell lines as models for ovarian cancer. Within this study we provide an extensive characterization of 39 of these cell lines on cellular and molecular level. The major strength of this study is that the analyses were all uniformly performed and therefore provide a rigorous comparison between the expression signatures of the cell lines. This uniformity was achieved by culturing all cell lines with the same medium and culture conditions, using the first passages after stable growth on the standardized culture conditions of the cell lines for all cellular and molecular analyses, and performing the molecular analysis of all cell lines simultaneously within one batch. We identified that the ‘COLO720E’ cell line did not match the reference short-tandem repeat (STR) profile and was excluded from further analyses.

The DNA mismatch repair system (MMR) is involved in repair of errors in these STR that occur during DNA synthesis. Therefore, defective MMR is associated with a large variability in the length of these repeats which is called micro-satellite instability (MSI). Indeed, the STR profiles of the MSI cell lines showed a significant lower concordance with their reference STR profiles. For instance, IGROV1 is one of the MSI cell lines that has been suggested by Korch et al. to have a variable STR profile with multiple peaks which may indicate cross-contamination [29]. Prof Korch kindly sent us their STR profiles generated from three IGROV1 DNA isolates including one obtained directly from the National Cancer Institute. Close examination of the peaks shows an average concordance of 74% between the three references and our profile (Table S3). Furthermore, the discordant peaks all show a shift (or extra peak) of only one repeat difference suggesting this could be due to ineffective repair of errors made during DNA synthesis. Thus, although STR profiling is very important to determine whether a cell line is contaminated or not, one should be careful with drawing a conclusion when the cell line has a defective MMR which can also cause multiple peaks in the STR profile.

Furthermore, MSI cell lines showed significantly more mutations per cell line then the non-MSI cell lines (median of 13 vs 2). Similarly, MSI colorectal cancer was shown to have ten times more non-synonymous mutations per tumour compared to non-MSI colorectal cancer [56]. This suggests that most of the mutations seen in MSI tumours as compared to non-MSI tumours are passenger events. It is therefore imperative when searching for driver mutations e.g. within the Cancer Genome Atlas (TCGA) project to take into account the MSI status of the tumours.

To be able to develop and test subtype-specific treatments in the correct model, it is imperative to know which histological subtype each cell line represents. To identify HGS cell line models, Domcke et al. analysed genomic data for 47 ovarian cancer cell lines from the CCLE collection [13] for genetic similarity to high-grade serous ovarian carcinomas from the TCGA data [4], [57]. In addition, Anglesio et al. used their predictive clinical algorithm COSP (based on nine immunohistochemistry markers) to predict the four major histological subtypes within their 32 cell lines [31]. We have tested similar assumptions for the 39 ovarian cancer cell lines described here based on the published histology and grade of the tumour of origin together with independent mutation analyses. We identified 20 non-serous and 19 serous cell lines including 14 high-grade serous and one low-grade serous. Comparison of our results with the two earlier studies [31], [57] shows similar prediction for HGS and some discrepancies for the non-serous cell lines. Interestingly, all three studies implicated widely used ovarian cancer cell lines like A2780, SKOV3 and IGROV1 as not being representative of the major HGS subtype but most likely of the endometrioid histological subtype. These data are very important for the correct interpretation of previous *in vitro* experiments using these lines and are vital for a rational experimental approach for future studies to develop subtype specific treatment approaches.

Based on differences in morphological and growth characteristics, we identified three *in vitro* morphological subtypes (i.e. Epithelial, Round and Spindle). These three morphological phenotypes were previously observed in multiple types of cancer cell lines including cervical, gastric, prostate, colorectal, lung and glioblastoma [59]–[70] and a panel of 54 breast cancer cell lines [58]. The *in vitro* Round morphology has been associated with the side population that shares characteristics with tumour stem cells of cervical and gastric tumour cell lines [59], [60] as well as with so-called amoeboid migration [61]–[65]. Epithelial to mesenchymal transition (EMT) is typically defined by the acquisition of a Spindle morphology and up-regulation of mesenchymal markers such as SNAIL, ZEB, TWIST, VIM, FN1 and CAV1 [66]–[70]. EMT is a process in which cell adhesion properties are altered and epithelial cells lose their cell polarity and gain migratory and invasive properties to become mesenchymal cells. Indeed, the genes up-regulated in the Spindle cell lines include 73 Extracellular matrix (ECM) component genes as defined by Hynes et al. [71]–[73] (i.e. 21% compared to 4–5% in Round and Epithelial) including several genes up-regulated during EMT (e.g. TGFBI, TGFBR2, TWIST1, ZEB1, ZEB2, VIM, FN1, CAV1)(Table S5).

These morphological subtypes are observed in cell lines from multiple tumour types and seem to associate with characteristics of putative clinical importance. It is therefore important to translate the *in vitro* morphological phenotype to a clinical phenotype. We performed an *in silico* analysis to determine whether the three *in vitro* identified morphological subtypes associate with clinical characteristics including prognosis as well as the molecular subtypes identified within ovarian carcinomas [11]. We normalized the cell line and carcinoma data separately to correct for differences between the two platforms used. However, we cannot predict how this reflects the biological differences such as the presence of stroma, fibroblasts and immune cells within tumour tissue which is not present in cell lines. Clustering of the cell lines together with the endometrioid and HGS carcinomas using the morphology associated genes showed two tumour clusters, 1) the Spindle-like tumours, and 2) the Epithelial and Round-like tumours.

The 152 genes over-expressed in the Spindle-like cluster (Figure 6, gene cluster A) associate with cell movement and invasion and include 44 Extracellular matrix (ECM) associated genes (e.g. ADAM12/19, 8 collagens, THBS1/2, LOX, TNC, MMP2) as well as genes overexpressed during Epithelial-to-Mesenchymal transition (EMT) (e.g. TGFBI, TGFBR2, TWIST1, VIM, FN1).

ECM remodelling as well as EMT have been associated with invasion and metastasis and thus a clinically more aggressive subtype. Indeed, the Spindle-like tumour cluster showed a significant enrichment for metastases as arrayed site, higher stage, sub-optimal debulking, and decreased progression-free survival. The increased expression of cell movement and invasion-associated genes could explain the metastasis, higher stage and worse prognosis observed.

We also found that the morphological tumour clusters were associated with the six molecular subtypes C1-C6 identified by Tothill et al. [11]. Cluster 1 contains all C1 (characterized by a reactive stroma signature and worse prognosis) and most C3 (LMP-like) carcinomas. The worse prognosis for the C1 subtype carcinomas previously observed by Tothill et al. thus explains the worse prognosis seen for the spindle-like cluster. Cluster 2 contains most C4, C5 (characterized by a mesenchymal signature) and C6 (low-grade endometrioid) tumours. The C2 carcinomas, characterized by an immune signature, are evenly divided over the two morphological tumour clusters which could be due to presence of immune cells in these tumours that are not present in cell lines. However, we did observe a gene cluster (cluster B) associated with immune response overexpressed in the C2 tumours as well as the Spindle cell lines suggesting that there may also be a cell autonomous effect, and it is not only the microenvironment that contributes to the immune signature.

Closer examination of the clustering showed that the Spindle cell lines have a very similar gene expression pattern as seen for the C1 (stromal) tumours, the Round cell lines resembled the C5-mesenchymal subtype and the Epithelial cell lines the C4 subtype (Figure 6). This could imply that the *in vitro* morphological phenotypes resemble the clinical molecular subtypes and could be used to select relevant cell lines for tumour subtype specific studies.

Interestingly, 61/152 genes overexpressed in the Spindle-like cluster (Figure 6, gene cluster A) overlap with the 292 genes up regulated in the C1 subtype explaining the association we see between the C1 subtype tumours and the Spindle cell lines. Furthermore, we see a small overlap between our *in vitro* morphology genes with the CLOVAR subtype signature of 100 genes [74] which is enriched for genes that are up in the CLOVAR Mesenchymal subtype that are also high in the Spindle subtype (12/19 overlapping genes). Both the C1 and the Mesenchymal subtype show a worse prognosis [11], [74] and an increase in (reactive) stromal gene expression that for the C1 subtype correlates with extensive desmoplasia and low tumour cell percentage. This suggests a greater contribution of the stromal compared to the epithelial tumour cells in these subtypes. Indeed, stromal fibroblasts play an important role in tumourigenesis and prognosis in several cancer types [75]–[77]. However, with our analyses of 39 cell lines we show that the spindle-shaped epithelial tumour cells also strongly contribute to the stromal signature underlining the importance of an epithelial tumour cell autonomous effect additive to the stromal cells.

The tumour microenvironment is also involved in multidrug resistance through a range of putative mechanisms, e.g. by decreasing drug concentrations through increased interstitial fluid pressure, 3D structure or by activating cellular pathways through ECM-Integrin binding, the so-called cell adhesion-mediated drug resistance (CAM-DR) [78]. In earlier studies we also observed an association between (TGFbeta-induced) ECM remodeling during EMT and chemotherapy resistance in ovarian cancer [18], [79] as well as endocrine therapy resistance in breast cancer [80]–[82].

This relation between mesenchymal subtype and therapy resistance was not observed for the Spindle cell lines. Possibly since within cell lines the cross-talk between the mesenchymal tumour cells and stroma is limited to the autonomously produced ECM and could therefore not fully mimic the clinical impact of the tumour microenvironment.

Although Spindle morphology itself was not associated with taxane response, one of the Spindle cell line characteristics, i.e. high CD44 expression, did associate with response to taxanes. The microtubules targeted by taxanes are indeed very important in cell movement which could explain why CD44-high Spindle cell lines were more sensitive. The literature suggests that CD44 high tumour cells display increased self-renewal, migration and therapy resistance and are suggested to be cancer initiating cells (CIC) [83]–[86]. Also in ovarian cancer, increased density of CD44 positive cells was associated with chemotherapy resistance [87], [88]. Interestingly, conjugates of Paclitaxel with the CD44 ligand hyaluronic acid were shown to be more effective in reducing tumour burden in implanted CD44 positive human ovarian carcinoma mouse models compared to free Paclitaxel [89], [90]. These data might suggest that by adding these conjugates to platinum-based chemotherapy, the CD44 positive (CIC) cells might be specifically targeted thereby decreasing CIC initiated relapses.

In summary, our study provides a uniformly generated data resource for a panel of 39 ovarian cancer cell lines. We identified histological as well as morphological subtypes which were associated with clinical pathological and molecular characteristics of ovarian carcinomas and prognosis. Therefore, this study is of considerable research value to those looking for better defined model systems with consistency to valid clinical phenotypes.

## Supporting Information

Figure S1 **Representative images of six examples of each of the three morphological subtypes to illustrate differences in cell shape, size and growth pattern between the morphological subtypes (50% confluence, 100x magnification).**
(TIF)

Figure S2 **CCNE1 amplification in three categories: A. no (n = 26, three examples shown), B. medium (2–3x MAD above the median, n = 2) and C. high amplification (>3x MAD above the median, n = 3).** The Y-axes represents the number of median absolute deviation (MADs) from the median coverage (Z-score), the maximum is given above each graph, each bar in the histogram represents an exon of the gene, blue bars are exons with >1.5 MAD above the median. D. CCNE1 mRNA expression (y-axes) relative to yes or no gene amplification (x-axes) (Mann-Whitney p<.001).
(TIF)

Figure S3 **Unsupervised clustering of the 26 unique ovarian cancer cell lines using the 80% most variable expressed mRNAs and microRNAs.** *Morphology*: E Epithelial, R Round, S Spindle, *Histology & Putative Histology*: S serous, HGS high-grade serous, LGS low-grade serous, E endometrioid, C clear cell, Mx mixed, M mucinous, *Time & treatment*: P primary disease, PU untreated primary disease, RU untreated relapsed disease, RP platinum treated relapsed disease, U untreated, (R)O (relapsed) disease that received other treatment, CRP at clinical resistance and platinum treated. *Protein markers*: bright red no expression (signal–to-noise ratio <5), light red low expression (signal–to-noise ratio 5–20), light green expression (signal–to-noise ratio 20–200), bright green high expression (signal–to-noise ratio >200), grey not determined. *Median response*: of eight therapeutics green to red scale sensitive to resistant. *Doubling time*: green less than one day, yellow 1–2days, orange >2days. *MSI* microsatellite instability. *# mutations*: total number of mutations. *Gene amplification*: orange amplified (2–3x SD above the median), red highly amplified (>3x SD above the median). *Heatmap*: Red colour high expression, Green colour low expression.
(TIF)

Table S1 **Background information on the origin of the cell lines based on literature.**
(XLSX)

Table S2 **Normalized expression data for 384 microRNAs in all 39 cell lines.**
(XLSX)

Table S3 **Short tandem repeat profiles of 39 cell lines measured with the PowerPlex 16 system (in bold) compared to the reference STR profiles from the cell line banks ECACC and ATCC (in italic) and STR profiles measured by Korch et al. (in regular font).** See Korch et al. [29] for the origin of these cell lines. *nd not determined*.
(XLSX)

Table S4 **Overview table of all mutations detected in the 39 cell lines measured by exon sequencing (SOLID and Tam-Seq), Sanger sequencing and SNApSHOT analysis.**
(XLSX)

Table S5 **Morphological subtype associated genes (n = 1141) and microRNAs (n = 18).** The table includes the corresponding cluster (High in Spindle, High in Round, High in Epithelial) as well as whether the gene is an extracellular matrix component according to Hynes et al. [71]–[73].
(XLSX)

File S1 **Supplemental methods.**
(DOCX)
