# Correction: Ovarian Cancer Cell Line Panel (OCCP): Clinical Importance of *In Vitro* Morphological Subtypes

**DOI:** 10.1371/journal.pone.0122284

**Published:** 2015-03-23

**Authors:** The PLOS ONE Staff

The following information is missing from the Funding section: EU-FP7- Ovarian cancer therapy, Innovative models Prolong Survival (EU-FP7- OCTIPS HEALTH-2011 279113)

## References

[1] Beaufort CM, Helmijr JCA, Piskorz AM, Hoogstraat M, Ruigrok-Ritstier K, Bessenlink N, et al (2014) Ovarian Cancer Cell Line Panel (OCCP): Clinical Importance of *In Vitro* Morphological Subtypes. PLoS ONE 9(9): e103988 doi: 10.1371/journal.pone.0103988 25230021 10.1371/journal.pone.0103988 PMC4167545

