# Supplementary figures and images for: Ovarian Cancer Cell Line Panel (OCCP): Clinical Importance of In Vitro Morphological Subtypes
Source: PLoS One. 2014 Sep 17;9(9):e103988. doi: 10.1371/journal.pone.0103988 (PMC4167545; doi:10.1371/journal.pone.0103988)

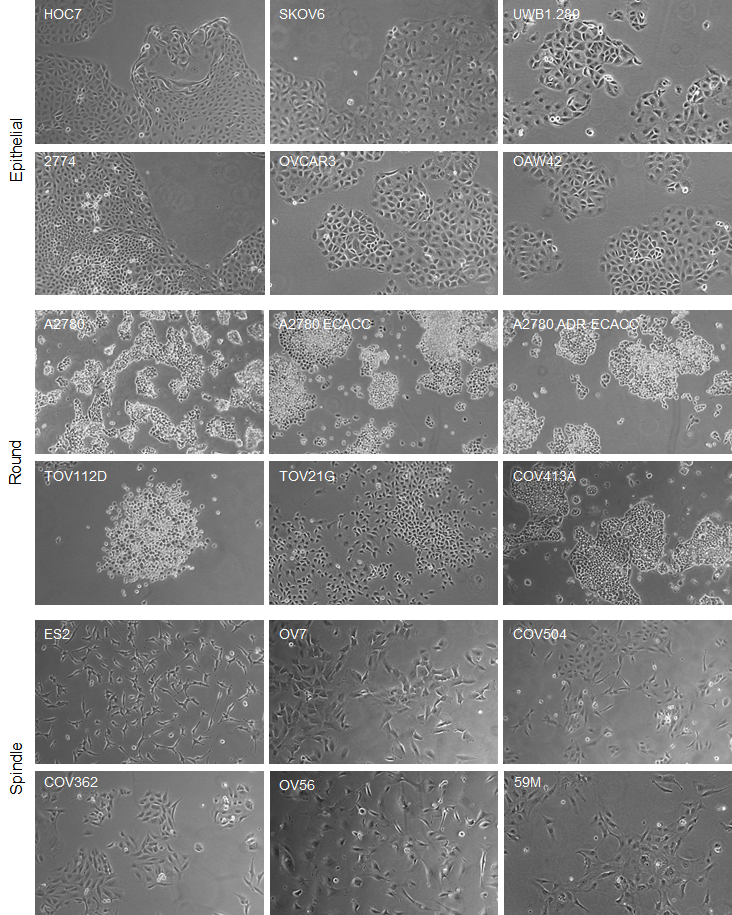

Supplement: Figure S1 — Representative images of six examples of each of the three morphological subtypes to illustrate differences in cell shape, size and growth pattern between the morphological subtypes (50% confluence, 100x magnification). (TIF) [file pone.0103988.s001.tif]

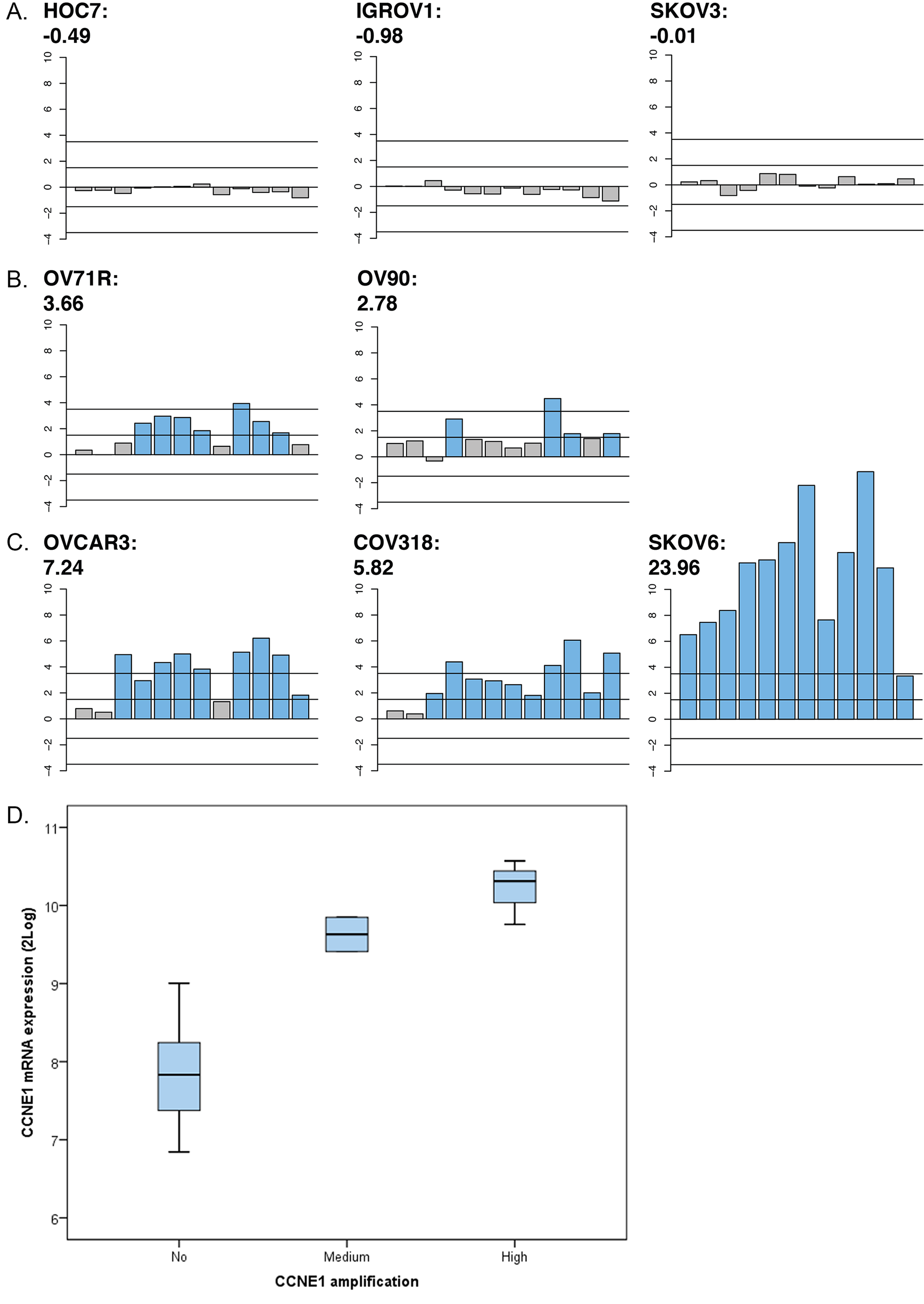

Supplement: Figure S2 — CCNE1 amplification in three categories: A. no (n = 26, three examples shown), B. medium (2–3x MAD above the median, n = 2) and C. high amplification (>3x MAD above the median, n = 3). The Y-axes represents the number of median absolute deviation (MADs) from the median coverage (Z-score), the maximum is given above each graph, each bar in the histogram represents an exon of the gene, blue bars are exons with >1.5 MAD above the median. D. CCNE1 mRNA expression (y-axes) relative to yes or no gene amplification (x-axes) (Mann-Whitney p<.001). (TIF) [file pone.0103988.s002.tif]

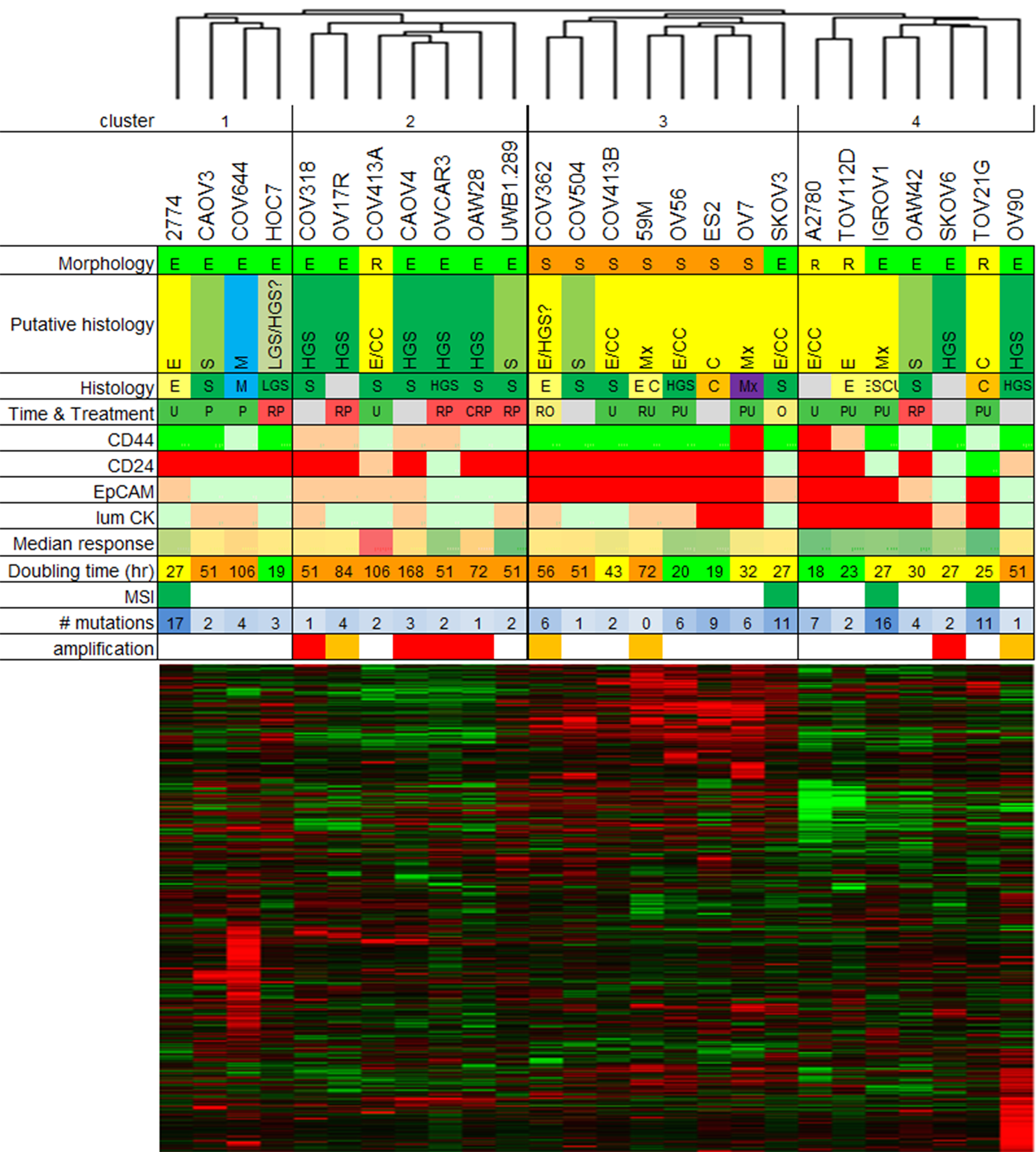

Supplement: Figure S3 — Unsupervised clustering of the 26 unique ovarian cancer cell lines using the 80% most variable expressed mRNAs and microRNAs. Morphology: E Epithelial, R Round, S Spindle, Histology & Putative Histology: S serous, HGS high-grade serous, LGS low-grade serous, E endometrioid, C clear cell, Mx mixed, M mucinous, Time & treatment: P primary disease, PU untreated primary disease, RU untreated relapsed disease, RP platinum treated relapsed disease, U untreated, (R)O (relapsed) disease that received other treatment, CRP at clinical resistance and platinum treated. Protein markers: bright red no expression (signal–to-noise ratio <5), light red low expression (signal–to-noise ratio 5–20), light green expression (signal–to-noise ratio 20–200), bright green high expression (signal–to-noise ratio >200), grey not determined. Median response: of eight therapeutics green to red scale sensitive to resistant. Doubling time: green less than one day, yellow 1–2days, orange >2days. MSI microsatellite instability. # mutations: total number of mutations. Gene amplification: orange amplified (2–3x SD above the median), red highly amplified (>3x SD above the median). Heatmap: Red colour high expression, Green colour low expression. (TIF) [file pone.0103988.s003.tif]
