# Supplementary material for: Ovarian Cancer Cell Line Panel (OCCP): Clinical Importance of In Vitro Morphological Subtypes
Source: PLoS One. 2014 Sep 17;9(9):e103988. doi: 10.1371/journal.pone.0103988 (PMC4167545; doi:10.1371/journal.pone.0103988)
Supplement: File S1 — Supplemental methods. (DOCX) [file pone.0103988.s009.docx]

**Supplemental Materials & methods**

*Growth curves and Drug sensitivity assay*

For the generation of growth curves, 24 concentrations of cells (range of 400.000 to 36 cells per well, 1.5 dilution factor) were plated in quadruplicate in a 96-well plate. After five days, the cells were incubated with MTT (3-(4,5-Dimethylthiazol-2-yl)-2,5-diphenyltetrazolium bromide, Sigma)(final concentration of 0.5 mg/ml) until cells turned blue. Plates were centrifuged to spin-down the non-adherent (viable) cells and medium was aspirated. DMSO with 0.25% NH3 was added to lyse the cells before measuring the OD at 570 nM. This assay was performed in duplicate for each cell line.

We choose the optimal number of cells from the two generated growth curves, i.e the highest number of seeded cells still showing linear growth after five days. The highest number of linear growing cells was selected because the cell number will decline when drugs are added in the drug sensitivity assays.

The response to chemotherapeutics was tested using a drug sensitivity MTT assay similar to the proliferation MTT assay. We seeded the selected number of cells (as described above) and added drugs to the media 24h later. Drug was added in 18 subsequent concentrations in quadruplicate with a dilution factor of 1.5 or 2 times. Medium without drug was added to the eight control wells. Cells were exposed to the drug for four days. Then MTT was added to the medium (final concentration of 0.5 mg/ml) and the cells were incubated until they turned blue. The plate was centrifuged to spin-down the non-adherent (viable) cells and medium was aspirated. DMSO with 0.25% NH3 was added to lyse the cells before measuring the OD at 570 nM.

The OD570 values of the drug treated cells were normalized by dividing them with the average OD570 value of the eight control wells without drug added. Model 108 (Inhibitory Effect Sigmoid Imax, C=0 at Imax, C=infinity at E0) and 107 (Inhibitory Effect Sigmoid E0, Effect C=0 at E0, C=infinity at 0) were used to fit a PD curve and calculate the 50% growth inhibition values (GI50) with error and 95% confidence intervals using Phoenix WinNonLin 1.1 software (Pharsight). Model 107 was used when the maximal effect at the highest dosage was 0, thus complete cell death.

*microRNA expression analysis*

# For microRNA expression analysis, TaqMan® Array Human MicroRNA A fluidic Cards v2.0 (Applied Biosystems) containing qRT-PCR assays to quantify 381 unique miRs were used according to the manufacturer's protocol. In brief, 400ng total RNA was used as input in a megaplex Reverse Transcription reaction (Megaplex™ RT primers Human Pool A v2.1 and TaqMan® microRNA Reverse Transcription Kit, Aplied Biosystems), which enables up to 381 microRNAs to be reverse transcribed in a single reaction. The product of six of these RT reactions was added to 896 µl PCR reaction mixture (TaqMan® Universal PCR Master Mix No AmpErase® UNG, Aplied Biosystems), and was added to the fluidic cards. Fluidic cards were run using a 384 well Taqman Low Density Array block in the 7900HT Fast Real-Time PCR system (Applied Biosystems,Nieuwekerk a/d IJssel, The Netherlands).

The expression data was normalized using the median Ct of all measured microRNAs as described by Vandesompele et al [1].

*Gene expression analysis*

One µg of high quality total RNA was used to synthesize cDNA using the GeneChip^®^ WT (Whole Transcript) Sense Target Labeling and Control Reagents Kit, according to the manufacturer’s protocol (Affymetrix, Inc., CA, USA). The sense cDNA was then fragmented by UDG (uracil DNA glycosylase) and APE 1 (apurinic/apyrimidinic endonuclease 1), and biotin-labeled with TdT (terminal deoxynucleotidyl transferase) using a GeneChip^®^ WT Terminal Labeling Kit. (Affymetrix, Inc., CA, USA). After the biotin-labeled sense target DNA was prepared, the sample was ready to hybridize to gene chip (The GeneChip^®^ Human Exon 1.0 ST array). Hybridization was performed using 5,5 µg of biotinylated target, which was incubated with a GeneChip^®^ Hybridization, Wash and Stain Kit and a GeneChip^®^ Fluidics Station 450 (Affymetrix, Inc., CA, USA). The arrays were scanned using a GeneChip^®^ Scanner 3000 (Affymetrix, Inc., CA, USA).

The acquired expression data was preprocessed using the Robust Multichip Analysis (RMA) algorithm within the Affymetrix^®^ Expression Console™ software that performs background correction, normalization and probe set summarization.

*SNaPshot mutation analysis*

### SNaPshot analysis was performed as described previously [2] using an Applied Biosystems SNaPshot Multiplex Kit. Reactions were performed in a volume of 9 μl containing 2.5 μl of SNaPshot Ready Multiplex Ready Reaction Mix, 1 × Big Dye sequencing buffer, 1 μl of probe mix for each position (probes used were published previously [3]and 1 μl Exo-SAP-it treated PCR product. Extension reactions were performed in a thermal cycler and consisted of 35 cycles of denaturation at 95°C for 10 sec and annealing/extension at 58.5°C for 40 sec. Labelled extension products were treated with shrimp alkaline phosphatase (1 unit per sample) then diluted 1 in 10. 1 μl of the diluted extension product was mixed with 9.8 μl of HiDi™ formamide and 0.2 μl of Genescan-120LIZ size standard. Products were denatured at 95°C for 5 minutes then separated using an ABI PRISM 3100 Genetic Analyzer with a 36 cm length capillary and POP-7™ polymer. Analysis was performed using GeneMapper 3.7 Software [4]. The nucleotide and corresponding amino acid changes evaluated were: for PIK3CA c.A3140G (H1047R), c.A3140T (H1047L), c.G1624A (E542K), c.G1633A (E545K), c.A1634G (E545G). For BRAF and the RAS genes the nucleotide positions evaluated were: BRAF (c.T1799 and c.A1801), for HRAS (c.G34, c.G35, c.G37, c.G38, c.C181, c.A182, c.G183), NRAS (c.G34, c.G35, c.G37, c.G38, c.A180, c.C181, c.A182 and c.A183), and KRAS (c.G34, c.G35, c.G37, c.G38, c.G57, c.C181, c.A182, c.A183).

*Mutation and amplification analyses with SOLiD exon sequencing*

The Automated Library Preparation and quality control was performed as follows. Processing of samples for sequencing on the SOLiD5500 sequencing platform (Life Technologies) is performed on a Sciclone NGS liquid handling robot (Perkin Elmer) and is based on the protocol for manual library preparation as published by Harakalova et al [5]. To allow the automated processing of samples, minor changes were implemented (detailed protocol used on the Sciclone NGS platform is available upon request). In brief, 600 ng DNA was fragmented using a Covaris LE220 (Duty Cycle 30%; PIP 500; Cycles per burst 200; for 500 seconds). Of the fragmented DNA 500ng was end-repaired using the End-It repair Kit (Epicentre) and subsequently purified using AMPure XP beads (Agencourt, Beckman Coulter). Blunt indexed adaptors (Life Technologies) are ligated to the blunted DNA, followed by 2 rounds of AMPure purifications. The ligation products are amplified by 7 rounds of PCR (5’ at 95°C; (15” 95 °C; 15” 62°C; 1’ 70°C for 7 cycles); 5’ 70°C) and the PCR reactions are purified using AMPure XP beads. Sheared, ligated and amplified products are analyzed on a LabChip GX chip (Perkin Elmer) according to standard protocols. For the amplified libraries, a total concentration per samples is determined by LabChip GX (Perkin Elmer), together with the concentration of the in-range fragments (150-300 bp).

Sure-Select enrichment for the Cancer Mini-Genome of 1971 cancer related genes was performed as follows. The in-range concentration is used to equimolarly pool samples (maximum of 16 per SureSelect enrichment), followed by an AMPure bead-based size-selection to isolate 150-300 bp products. The pooled and size-selected sample is subsequently enriched, with a minimum of 500ng, using a custom designed SureSelect enrichment kit containing 1971 cancer related genes (based on our previous finding [6]. The enrichment is performed in a multiplexed fashion of up to 16 samples per reaction (based on Nijman et al [7]). After the enrichment procedure, the sample is amplified by 12 rounds of PCR, purified and the concentration is determined by Qubit (Invitrogen). On average 4.2 ng is used as input for the EZ-bead system (E120 system) prior to SOLiD 5500xl sequencing. The EZ-bead processing and SOLiD sequencing is performed according to manufacturers protocol (Life Technologies).

Data were mapped to the reference genome (GRCh37/hg19) using BWA (-c -l 25 -k 2 -n 1). SNP and indel calling was done using a custom analysis pipeline that identifies mutations with a non-reference allele frequency larger than 15%, a coverage of at least 10× and presence in minimally 3 independent reads after filtering out ambiguously mapped reads and bases with a quality value < 25.

The 53 genes mutated or amplified in ovarian cancer analyzed are: TP53, RB1, NF1, CDK12, PIK3CA, KRAS , HRAS, NRAS, BRAF, ARID1A, CTNNB1, PTEN, PPP2R1A, EGFR, homologous recombination repair (BRCA1, BRCA2, BARD1, BRIP1, CHK2, PALB2, RAD50, RAD51), WNT/ b-catenin pathway (APC, CHD8, CREBBP, CSNK2A1, CTBP2, CUL1, DAAM2, EP300, FZD1, FZD3, LEF1, LRP5, MAP3K7, MMP7, NFAT5, NFATC3, PPP3R1, PRICKLE1, PRICKLE2, PRKACG, ROCK1, ROCK2, SENP2, SMAD4, TCF7L2, VANGL1, VANGL2) and amplification of CCNE1, MYC (8q24), TPX2 (20q11) and ERBB2. For seven genes (CTBP2, DAAM2, FZD1, LRP5, VANGL2, TP53, ERBB2) the average coverage was below 20 for more than 50% of the cell lines increasing the chance of missing mutations in these cell lines.

For the analysis of gene amplification, robust Z-scores were calculated per exon by subtracting the coverage from the median coverage of that exon and dividing this by the median Absolute deviation (MAD), as described by Iglewicz and Hoaglin [8]. A gene is amplified with a Z-score greater than 2 (thus >2 times the MAD above the median) and highly amplified if greater than 3 (thus >3 times the MAD above the median).

1. Mestdagh, P., et al., *A novel and universal method for microRNA RT-qPCR data normalization.* Genome Biol, 2009. **10**(6): p. R64.

2. Ramirez-Ardila, D.E., et al., *Hotspot mutations in PIK3CA associate with first-line treatment outcome for aromatase inhibitors but not for tamoxifen.* Breast Cancer Res Treat, 2013. **139**(1): p. 39-49.

3. Lurkin, I., et al., *Two multiplex assays that simultaneously identify 22 possible mutation sites in the KRAS, BRAF, NRAS and PIK3CA genes.* PLoS One, 2010. **5**(1): p. e8802.

4. Kompier, L.C., et al., *FGFR3, HRAS, KRAS, NRAS and PIK3CA mutations in bladder cancer and their potential as biomarkers for surveillance and therapy.* PLoS One, 2010. **5**(11): p. e13821.

5. Harakalova, M., et al., *Multiplexed array-based and in-solution genomic enrichment for flexible and cost-effective targeted next-generation sequencing.* Nat Protoc, 2011. **6**(12): p. 1870-86.

6. Vermaat, J.S., et al., *Primary colorectal cancers and their subsequent hepatic metastases are genetically different: implications for selection of patients for targeted treatment.* Clin Cancer Res, 2012. **18**(3): p. 688-99.

7. Nijman, I.J., et al., *Mutation discovery by targeted genomic enrichment of multiplexed barcoded samples.* Nat Methods, 2010. **7**(11): p. 913-5.

8. Iglewicz, B. and D. Hoaglin, *How to Detect and Handle Outliers*. 1993, Milwaukee, Wis: ASQC Quality Press.

9. Sieuwerts, A.M., et al., *Anti-epithelial cell adhesion molecule antibodies and the detection of circulating normal-like breast tumor cells.* J Natl Cancer Inst, 2009. **101**(1): p. 61-6.
